# Processing Characteristics of Ultra-Precision Cutting of 4H-SiC Wafers by Dicing Blade

**DOI:** 10.3390/mi17020187

**Published:** 2026-01-30

**Authors:** Yufang Wang, Zhixiong Li, Fengjun Chen, Zhiqiang Xu

**Affiliations:** 1 College of Intelligent Manufacturing, Hunan Open University, Changsha 410004, China; wangyufang@hnou.edu.cn; 2 School of Mechanical Engineering and Mechanics, Xiangtan University, Xiangtan 411105, China; 18673281130@163.com; 3 School of Robot Engineering, Wenzhou University of Technology, Wenzhou 325000, China; abccfj@126.com

**Keywords:** dicing blade, 4H-SiC, chipping, process parameters, material removal

## Abstract

Dicing is an important process in the packaging segment of the semiconductor manufacturing process, and due to the high hardness and brittleness of 4H-SiC wafers, they are prone to crack propagation and severe chipping during the dicing process. To reduce chipping defects, this study investigates the effects of key process parameters on the chipping behavior of 4H-SiC wafers, as well as the associated chipping formation and material removal mechanisms during dicing. Firstly, a spindle current measurement scheme was designed to indirectly reflect changes in grinding force during the cutting process, and the change in the cutting process in a single pass was analyzed. Secondly, experiments controlling single-factor variables were designed to explore the influence of laws of process parameters, including depth of cut, spindle speed, feed speed, and the dicing blade parameter, abrasive grain size, on the quality of chipping, and the optimal process parameters were obtained. Thirdly, the morphology of the 4H-SiC cutting contact arc area, front–back chipping, and sidewalls was analyzed in order to investigate the chipping formation and material removal mechanism. This study contributes to a fundamental understanding of material removal mechanisms during the cutting of 4H-SiC wafers and other advanced semiconductor materials and provides guidance for optimizing cutting process parameters.

## 1. Introduction

Silicon carbide (4H-SiC), as a typical third-generation semiconductor material, has excellent characteristics such as wide bandwidth, high thermal conductivity, high critical breakdown electric field, high carrier mobility, and chemical inertness [1]. Therefore, semiconductor devices made from 4H-SiC are suitable for high-temperature, high-voltage, high-frequency, and high-power applications, as well as environments with strong radiation and harsh chemical conditions [2], and are widely used in power electronics, photovoltaics, electric vehicles, rail transportation, and related fields [3,4]. After the silicon carbide wafers have been fabricated and electrically tested, the silicon carbide chips need to be separated by a dicing process. The current methods of 4H-SiC wafer dicing mainly include laser dicing and dicing blade cutting. Laser dicing is a promising method for separating 4H-SiC chips with high efficiency and quality, and mainly includes laser ablation dicing and stealth laser dicing [5,6,7] and thermal laser separation [5,8,9]. However, laser ablation is limited to wafers of about 150 μm thickness, stealth dicing requires a dicing channel without pre-existing process control layers or metal structures, and an additional fragmentation process is required to separate the chips. Thermal laser separation of the chip sidewalls is of excellent quality and free from fragmentation and crystal defects, but the equipment cost is high, and the dicing process is complex [10]. Therefore, compared to laser cutting, dicing blade cutting is still an important process with excellent sidewall quality, relatively low equipment costs, and no thermal damage, and can play an important role in composite cutting processes for cutting and manufacturing special microstructures [11,12].

As 4H-SiC is a typically hard and brittle material with a Mohs hardness of up to 9.5, compared to 10 for diamond and 7 for silicon, this increases the difficulty of mechanical dicing. During the cutting process, the workpiece is susceptible to crack propagation and chipping on the top-back side of the chip during the cutting process, reducing the chip’s strength and affecting the performance of its use. Processing difficulties have also been reported in various processes, such as grinding and polishing of 4H-SiC [13,14]. In addition, recent studies have shown that diamond cutting and ultra-precision machining of 4H-SiC also face significant challenges related to brittle fracture, tool wear, and surface integrity [15,16]. In addition, 4H-SiC long crystal efficiency is low, the growth rate is about 1–2 mm/h, compared to the silicon long crystal rate, which is two orders of magnitude lower, and the long crystal process is prone to defects, which makes the 4H-SiC substrate manufacturing cost high [17]. Therefore, the study of the influence of key process parameters on the quality of 4H-SiC wafers during the dicing process, as well as the chipping formation and material removal mechanism, can help to optimize the dicing process and improve the yield of the chip, and reduce the economic losses caused by the dicing process link.

It is well known that the dicing blade must be used with suitably selected dicing process parameters to achieve the best cutting performance of the dicing blade, in order to reduce chipping defects generated during the cutting process. Many researchers have investigated the cutting quality of 4H-SiC under relevant process parameters based on the use of different types of dicing blades [18,19,20,21,22,23]. However, most of these studies mainly focused on resin-bonded or metal-bonded blades and emphasized parameter optimization, while detailed process-resolved analysis and chipping mechanism differentiation for hub-type ultra-thin electroplated blades are still scarce. FUJITA et al. [19] prepared a 50 μm thick, high-stiffness, high-hardness polycrystalline diamond dicing blade (PCD) to achieve ductile mode machining of silicon carbide substrates. Chu et al. [20] investigated the method of applying a metallic glass coating on the surface of an Fe-Co-Sn sintered diamond dicing blade, which resulted in a low coefficient of friction (CoF) of the dicing blade and reduced the size and number of 4H-SiC chipping. Xie et al. [21] prepared 4H-SiC microchannels using a thin resin-bonded diamond grinding wheel, and comprehensively investigated the effects of grinding process parameters, such as wheel rotational speed, feed rate, and depth of grind, as well as dicing blade parameters, such as grit size and thickness, on microchannel geometry and surface roughness. Yuan et al. [22] compared the chipping quality of 4H-SiC cut by a resin-bonded dicing blade and a metal-bonded dicing blade under different process parameters and showed that the resin-bonded dicing blade has better cutting performance.

In the area of material removal mechanisms regarding silicon carbide cutting, fewer basic research reports were found. Since the physics of dicing blade cutting is essentially a mechanical grinding process, and material removal is the result of the interference of the individual abrasive cutting edges with the workpiece, it is possible to benefit from experimental studies on indentation scribing of silicon carbide, as well as from studies on the grinding process of silicon carbide [24]. Meng et al. [25] performed nano-scratching experiments on 6H-SiC using a Berkovich indenter and concluded that the plastic deformation mechanism of 6H-SiC during ductile domain processing is likely to be a combination of dislocation activity and phase transformation. Tang et al. [26] designed variable load nano-scratch experiments on 4H-SiC and found that radial cracks develop on the surface, intermediate cracks and transverse cracks appear on the subsurface as the load increases, and the extension and interaction of the three types of cracks lead to brittle fracture and debris accumulation in the material. Wang et al. [27] investigated single and double scratch experiments on 4H-SiC, which showed that the scratch interval had a significant effect on the material removal mechanism. Agarwal et al. [28] reported the results of surface and subsurface experiments on milled 4H-SiC under different processing conditions and concluded that internal displacements caused by microcracks along grain boundaries contribute to material removal. Tsukimoto et al. [16] evaluated the grinding-induced damage layer in 4H-SiC wafers by combining HR-EBSD analysis with TEM observation, and concluded that the delamination and inhomogeneous distribution of elastic strain in the grinding-damaged layer were closely related to the distribution of lattice defects. Huang et al. [29] proposed an MD simulation model to explain the material removal mechanism of 4H-SiC in scratch tests. And phase transitions, dislocations, and stacking faults were observed in the damaged layer. Wu et al. [30,31] analyzed the plastic deformation of 6H-SiC during nano-grinding using MD simulations, taking into account the effects of diamond grit position mode, size, and protrusion height. Grain position mode significantly affects the average grinding force, groove profile, and defect morphology. Grain size and protrusion height influenced the transformation of adhesion, plowing, and cutting mechanisms.

Although hub-type dicing blades have been widely used in semiconductor manufacturing, systematic studies focusing on ultra-thin electroplated hub-type diamond blades applied to 4H-SiC wafers, especially on the C-face, are still limited. In addition, most existing studies mainly report final cutting quality or average cutting forces, while the dynamic cutting behavior during a single dicing pass and the distinct mechanisms of front-side and backside chipping remain insufficiently understood.

Therefore, this study aims to (i) analyze the full single-channel dicing process of 4H-SiC using spindle current segmentation to reveal the dynamic cutting characteristics; (ii) systematically investigate the influence of key process parameters and abrasive grain size on front-side and backside chipping; and (iii) clarify the chipping formation and material removal mechanisms by combining contact arc zone observation, sidewall morphology analysis, and undeformed chip thickness modeling. The results provide new insights into the ductile–brittle removal behavior of 4H-SiC during ultra-precision dicing and offer guidance for process optimization.

## 2. Laboratory Equipment and Process Experiments

**Figure 1:**
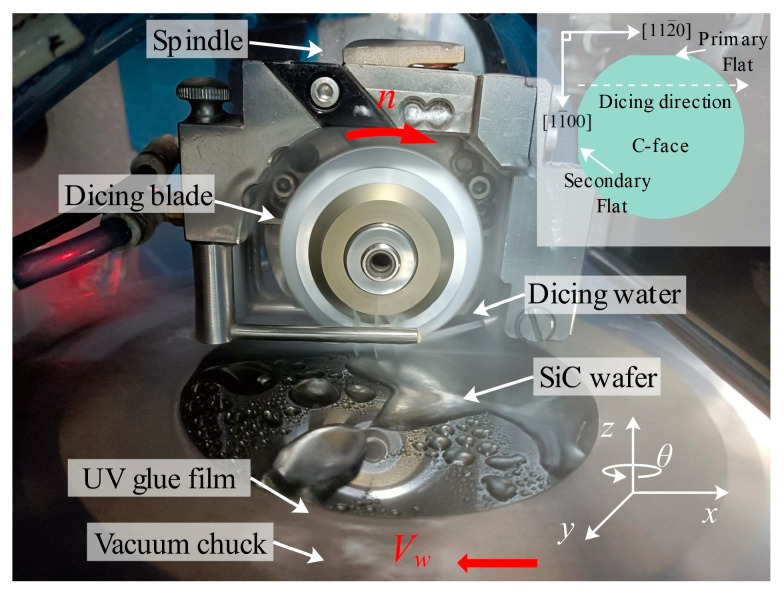
Diagram of the dicing experiment setup.

The dicing equipment used in the experiments was an SD1212A (HEFEI AIKAIRUISI INTELLIGENT EQUIPMENT CO., LTD. Hefei, China) model. Its spindle power is 1.8 kW, the maximum spindle speed is 60,000 rpm, table flatness ±0.005 mm/300 mm, dicing experimental device is shown in Figure 1. The cut workpiece is a 4-inch P-type 4H-SiC with a thickness of 360 ± 15 μm. The carrier film used is UV film with a total thickness of 0.160 mm and a glue thickness of about 10 μm. The dicing blade is an ultra-thin electroplated diamond dicing blade produced by SSTech, Ho Chi Minh, Vietnam, model 3000-R-70 DCB, with the parameters shown in Table 1. The base part of this dicing blade is an aluminum alloy hub, and the blade part is a mixture of nickel and diamond, which is suitable for precision cutting of semiconductor materials such as Ge, Si, SiC, GaN, GaAs, and InP. Compared with other types of dicing blades, the thickness of this dicing blade is thinner, which reduces the loss of material from the cut wafer; the abrasive grain size used is smaller, which helps to reduce chipping; and the electroplated nickel bond has higher hardness and wear resistance, and a long service life.

In this study, the spindle inverter current was used as an indirect indicator of the grinding force. Under the constant-speed control mode of the inverter, variations in spindle current reflect changes in spindle torque and mechanical load. Although no direct force calibration was conducted, relative changes in spindle current under identical control conditions are considered effective for comparing cutting states and process trends.

**Figure 2:**
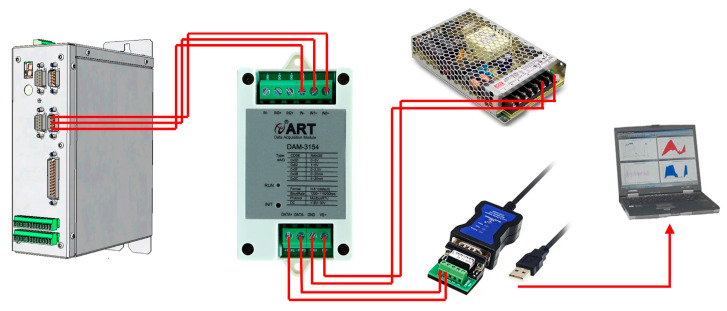
Schematic diagram for measuring spindle inverter current.

**Figure 3:**
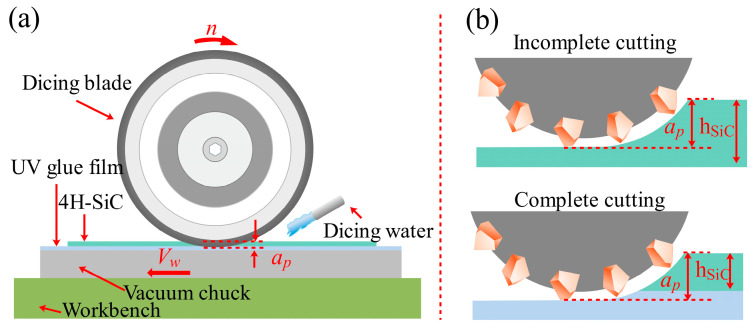
(**a**) Schematic diagram of dicing blade cutting principle, (**b**) schematic diagram of dicing blade incomplete cutting and full cutting.

In order to facilitate real-time monitoring of the changes that occur in the grinding force signal during the cutting process, the DAM-3154 analog acquisition card is used to collect the voltage signal from the inverter analog port, which indirectly reflects changes in grinding force through the spindle inverter current. The current signal was sampled at approximately 20 Hz, and the schematic diagram for measuring the spindle current is shown in Figure 2. The key process parameters of the cutting experiment are listed in Table 2, where *a_p_* represents the depth of cut, *V_w_* denotes the feed speed of the workpiece, and *n* is the spindle rotational speed. These parameters were selected to investigate their influence on the chipping characteristics and spindle current variation. The experiments were conducted in two distinct modes: “incomplete cutting” and “complete cutting”. Incomplete cutting (also known as grooving) refers to the process where the depth of cut (*a_p_*) is set smaller than the wafer thickness (360 ± 15 μm), leaving a residual layer of material at the bottom. This mode is primarily used to analyze the cutting behavior inside the bulk material without the interference of bottom-edge fracturing. Conversely, complete cutting involves dicing through the entire wafer thickness and penetrating into the UV tape to achieve die separation. The schematic diagram of dicing blade cutting is shown in Figure 3a, and the schematic diagrams of incomplete cutting and complete cutting are shown in Figure 3b.

Before cutting the workpieces, the dicing blade was mechanically sharpened to open the cutting edges and enhance its cutting performance. A precut board specifically designed for this type of blade, manufactured by SSTech Company, was used as the sharpening tool. The abrasive grain size of the dicing blade was 3000 mesh, while the precut board had a mesh size of 2000 mesh. After mechanical sharpening, the blade was further used to cut approximately 1 m of silicon carbide material as a pre-cutting process, aiming to stabilize the surface morphology of the dicing blade and promote uniform exposure of abrasive grains under actual cutting conditions.

Subsequently, the silicon carbide wafer was diced into rectangular specimens with dimensions of 35 mm × 20 mm. Cutting experiments were then carried out using different groups of process parameters. For each parameter group, two cutting channels were performed, and the spacing between adjacent cutting channels was set to 3 mm. Considering the anisotropy of 4H-SiC, all experiments were conducted on the C-face (0001) of the wafer, with the cutting direction aligned along the crystallographic direction $\left[11\bar{2}0\right]$. The cutting direction was kept constant throughout the experiments to avoid the influence of orientation-dependent properties.

**Figure 4:**
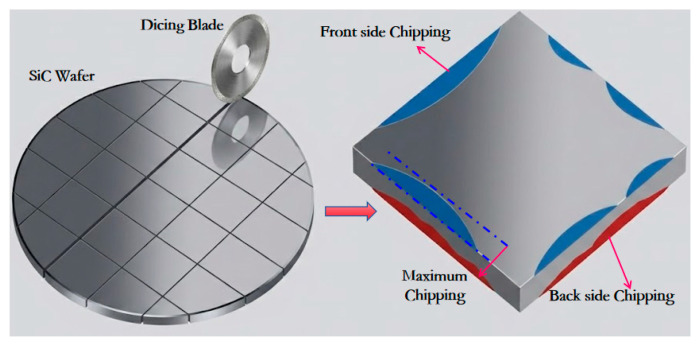
Schematic of chipping measurement geometry.

Furthermore, after completing the cutting experiments using the 3000-mesh dicing blade, additional experiments were conducted to investigate the influence of abrasive grain size on cutting quality. Dicing blades with grit sizes of 3500, 2500, and 2000 mesh from the same series were employed to perform cutting tests on the samples under identical experimental conditions. Current data were recorded for each set of process parameters during the experiment, and the average value of the two sets of current data was used to evaluate the spindle current. To ensure the reproducibility of the experimental results, each set of process parameters was repeated on two independent dicing channels. For quantitative evaluation, chipping measurements were collected from five randomly selected locations along each channel, resulting in a total of 10 measurement points per condition. The average value of these local maxima was calculated to represent the chipping quality, and the standard deviation was recorded to reflect the process stability. In addition, chipping was evaluated at multiple positions along each channel, further enhancing the statistical representativeness of the results. Tool wear was not treated as an independent variable in this study. To minimize its influence on cutting quality, the dicing blade was mechanically dressed prior to the experiments, and the total cutting distance for each blade was limited. Therefore, the reported results mainly reflect the influence of process parameters rather than progressive tool wear. A Zeiss (Oberkochen, Germany) microscope (Axio Lab A1) was used to observe the morphology of the cut channel after 4H-SiC cutting. The maximum value of top-back chipping was randomly measured at five different positions of each cut channel, and the mean value was taken to evaluate the quality of the cut, as shown in Figure 4. Chipping was defined as the lateral distance from the nominal edge of the cut channel to the farthest edge of material break-out. For each cutting channel, chipping was measured at five randomly selected positions. At each position, the maximum local chipping width was recorded, and the average value of these five local maxima was used to evaluate the chipping quality.

The diagram illustrates the definition of front-side and backside chipping from a top-down perspective (viewing perpendicular to the C-face of the wafer). The maximum lateral distance from the theoretical cutting line to the fracture edge is recorded as the chipping size. Note that in the optical micrographs presented in this study (e.g., Figures 6, 8, and 10), the dark central band represents the dicing kerf (groove bottom), while the irregular jagged boundaries on both sides represent the chipping areas.

In order to obtain key information about the material removal mechanism of 4H-SiC cutting, it can be characterized by observing the surface properties of the cutting contact arc zone, in addition to the surface characteristics of the sidewalls, which should not be neglected. Therefore, in the case that the dicing blade completely cuts into the workpiece and has not yet cut out the workpiece, the *Z*-axis direction of lifting the knife to stop cutting is carried out in order to obtain the cutting contact arc zone. The process parameters used were the best set of parameters for cut quality in the above experiments. A Keyence VK-X3000 Shape Measurement Laser Microscope was used to observe the surface characteristics of the cut contact arc area, as well as the sidewalls, and to measure the surface roughness. Sidewall roughness was measured using a Keyence VK-X3000 laser scanning microscope with a 100× objective. The Ra values were extracted from defect-free local regions of the sidewall using standard Gaussian filtering and a defined evaluation length. Therefore, the reported Ra represents local surface roughness rather than an average over the entire cutting depth. Throughout the experiment, the ambient temperature was 12.6 °C, the dicing water used was pure water with a water temperature of 9.2 °C, the flow rate of the front cutting water was 3 L/min, the flow rate of the side cutting water was 1.5 L/min, and the spray angle was not changed.

## 3. Experimental Results and Discussion

### 3.1. Single-Channel Cutting Process Analysis

**Figure 5:**
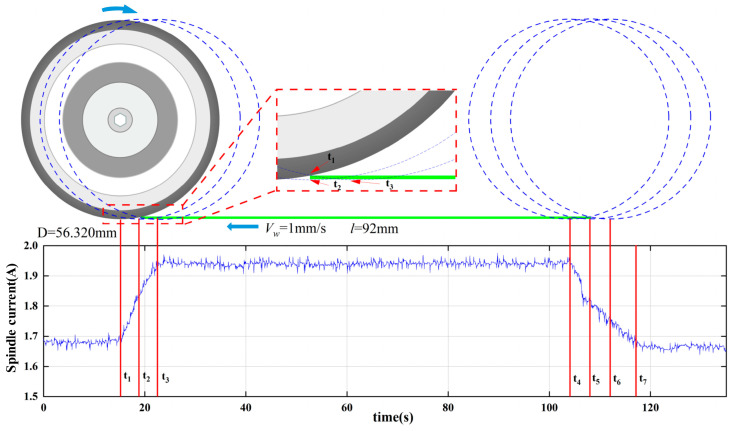
Spindle current acquisition results for single-channel cutting of silicon carbide.

Figure 5 shows the results of spindle current acquisition for 4H-SiC single-channel cutting under full cut conditions. The length of this cutting channel is 92 mm, and the process parameters of dicing are spindle speed of 30,000 rpm, feed rate of 1 mm/s, and depth of cut of 390 μm. A total of seven critical cutting nodes from t1 to t7 are marked in Figure 5. Before time t1, the dicing blade is not in contact with the workpiece. The average spindle current is about 1.68 A, which is mainly attributed to the drag force exerted by the cutting water on the high-speed rotating dicing blade. For t1 time, the dicing blade and the workpiece are just in contact, whereas for t2 time, the dicing blade’s lowest point just cuts into the workpiece. For the t1–t2 time period, with the gradual increase in the area of the cutting arc of the contact zone, the spindle current increases linearly. For t3, the dicing blade completely cut into the workpiece. For t3, the blade completely cut into the workpiece at the moment; for the t2–t3 stage, the cutting contact arc area does not change, but the spindle current is still a linear growth trend. The reason for this phenomenon is that the side of the blade and the workpiece interference area increase, and as the side of the blade and the workpiece interference area increase, the spindle current is also increased accordingly. The degree of interference between the sidewalls of the dicing blade and the workpiece may be related to the elastic bending of the dicing blade when it cuts high-hardness silicon carbide. In addition, the debris generated by the cutting may also be embedded in the gap between the sidewalls of the dicing blade and the workpiece, which increases the loading of the dicing blade. As for the decrease in the rate of increase compared to the rate of increase in the t1–t2 phase, it is due to the fact that the removal of the workpiece material occurs mainly through the interference of the abrasive particles on the end face of the dicing blade with the workpiece.

At the t3–t4 stage, the contact state between the blade and the workpiece does not change, and the spindle current remains relatively stable. At the t4–t5 stage, the blade slowly cuts out the workpiece, the area of the cutting contact arc gradually decreases, and the spindle current also decreases. t5 is the moment when the blade cuts out the workpiece at its lowest point, and at this time, the material of the cutting channel has been completely removed; however, at the t5–t6 stage, the spindle current is still slowly decreasing, which is also the result of the interference between the side of the blade and the workpiece before it is completely cut out. This is also due to the fact that the blade has not completely cut out the workpiece, and the side of the blade is interfering with the workpiece. At t6, the blade has completely cut out the workpiece, but the spindle current is not restored to 1.68 A until t7. This is due to the fact that the height of the workpiece is 360 μm, and the cutting water meets the workpiece, which leads to a change in the flow trajectory of the cutting water and an increase in the loading of the blade.

According to the above analysis, when the dicing blade completely cuts into the workpiece, the average value of the spindle current is about 1.95 A, compared with the current in the stage of not cutting the workpiece, the current increases by 0.27 A. In addition, we measured the current in the state of turning off the cutting water without cutting the workpiece at a spindle speed of 30,000 rpm, and the average value of the current is 0.49 A. Compared with the case of turning on the cutting water and not cutting the workpiece, the spindle current has changed by 1.19 A. This indicates that the power consumption of the spindle during the cutting process is nearly 60% for overcoming the resistance of the cutting water to the dicing blade and 15% for the removal of material from the workpiece. In addition, it is more reasonable to use the stage when the dicing blade completely cuts into and is about to cut out the workpiece, i.e., the stage t3–t4, and calculate the average value of the spindle current in this stage to evaluate the cutting state, which is adopted for the calculation of the average value of the current in this thesis.

### 3.2. Influence of Process Parameters on the Quality of Chipping

#### 3.2.1. Effect of Depth of Cut on Chipping Quality

**Figure 6:**
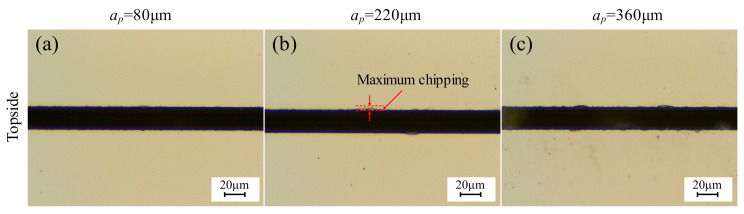
Topside chipping micrographs of 4H-SiC cut by dicing blade at different depths of cut, incomplete cuts. (**a**) *a_p_* = 80 μm; (**b**) *a_p_* = 220 μm; (**c**) *a_p_* = 360 μm (*V_w_* = 1 mm/s, *n* = 30,000 rpm).

**Figure 7:**
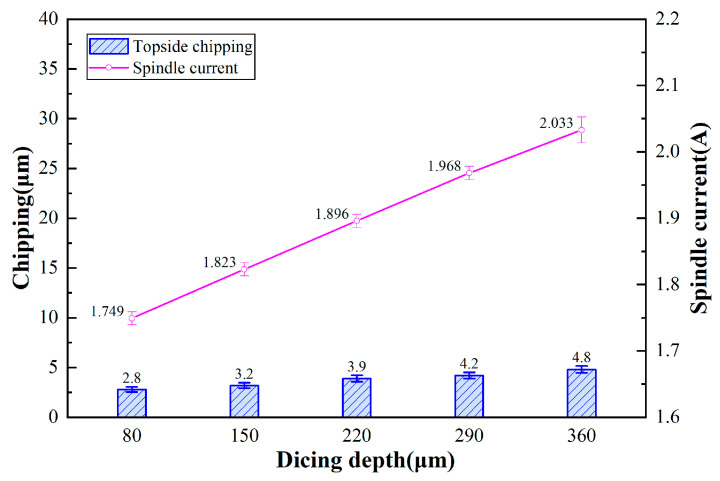
Effect of depth on chipping of 4H-SiC and spindle current, incomplete cuts.

Figure 6 presents the topside chipping micrographs of 4H-SiC cut by the dicing blade at different depths of cut (incomplete cuts). Detailed observation reveals the specific features of the cut geometry: the images capture the top view of the dicing trench. The dark linear band spanning the center of each micrograph (approximately 20–30 μm wide) corresponds to the dicing kerf (groove bottom) formed by the blade removal. The irregular jagged edges along both sides of this dark band represent the chipping areas. The quantitative chipping data presented in Figure 7 (and similarly in Figures 9, 11, 13, and 15) were derived from the statistical analysis of the optical micrographs corresponding to each experimental condition. Specifically, for each data point plotted in the graphs, chipping width measurements were taken from five randomly selected locations along the dicing channel in the micrographs. The average value of these measurements was calculated to represent the chipping quality, and the standard deviation was plotted as error bars to indicate the variability. Figure 7 shows the effect of depth on chipping of 4H-SiC and spindle current, incomplete cuts. As the depth of cut increases, the volume of material removed by a single grit cut increases, which increases the loading of the dicing blade, the spindle current becomes linearly larger, and the topside chipping tends to grow.

**Figure 8:**
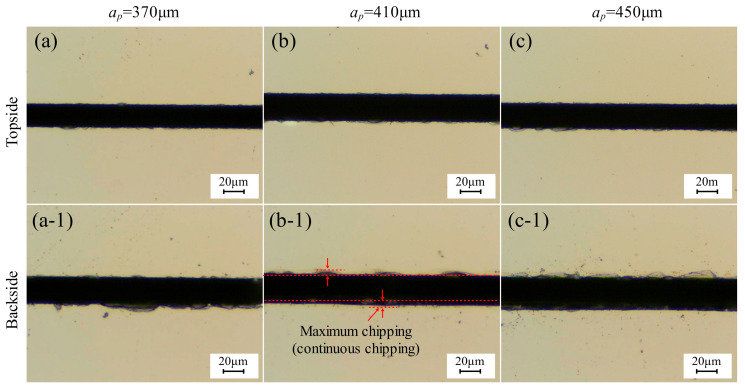
Chipping micrographs of 4H-SiC cut by dicing blade at different cutting depths: (**a**–**c**) front, (**a-1**–**c-1**) back (*V_w_* = 1 mm/s, *n* = 30,000 rpm).

**Figure 9:**
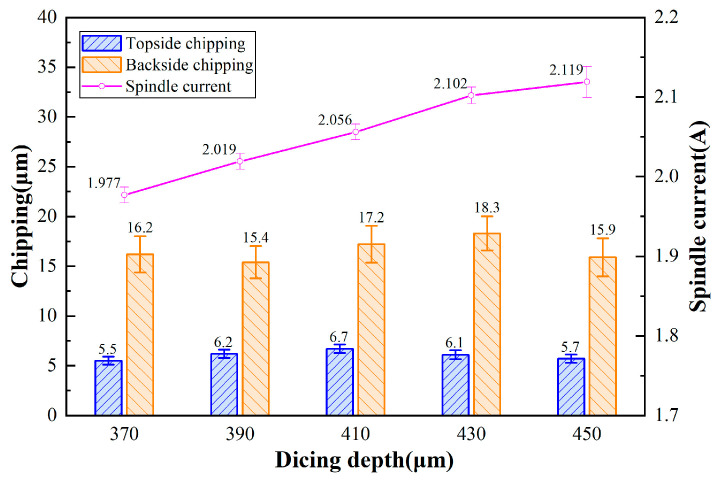
Effect of depth on chipping of 4H-SiC and spindle current (*V_w_* = 1 mm/s, *n* = 30,000 rpm).

Figure 8 displays the chipping micrographs of 4H-SiC cut by the dicing blade at full cut, with different depths of cut. Figure 9 shows the effect of depth on the chipping of 4H-SiC and current. The spindle current increases with increasing depth of cut. However, it was found that the spindle current was less than the current at 360 μm in the incomplete cut condition for the depth of cut of 370 μm and 390 μm, which may be due to the fact that in the cut-off condition, the generated debris can be easily discharged from the bottom of the workpiece, and the cutting water is more easily accessed to the cutting area, which reduces the load on the dicing blade. At depths of cut greater than 390 μm, the spindle current exceeds 2.033 A, which is mainly attributed to the increased depth of cut into the film, increasing the load on the dicing blade. The top-back chipping does not change significantly with increasing depth of cut, which is mainly due to the fact that the total volume of the removed workpiece material does not change under full cutting conditions. Observing Figure 8(b-1), the continuity chipping occurs on one side, and the other side is intermittent chipping, and the chipping on the two sides shows different characteristics, which is related to the elastic bending that occurs during the cutting process of the dicing blade, because the ultra-thin dicing blade may have a lack of rigidity during the cutting process of the 360 μm thick SiC wafers, and it is also related to the fact that the UV film possesses a certain degree of elasticity, and the cutting under the complete cut-off condition generates a lateral force that will cause the separated chip to be slightly displaced to one side.

#### 3.2.2. Effect of Feed Speed of Cut on Chipping Quality

**Figure 10:**
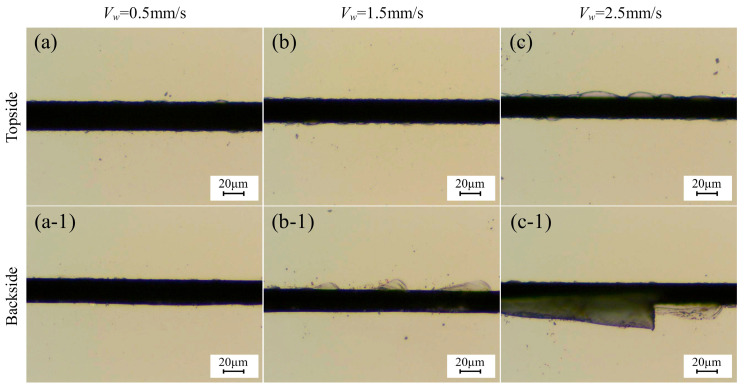
Chipping micrographs of 4H-SiC cut by dicing blade at different feed speeds: (**a**–**c**) front, (**a-1**–**c-1**) back (*a_p_* = 390 μm, *n* = 30,000 rpm).

**Figure 11:**
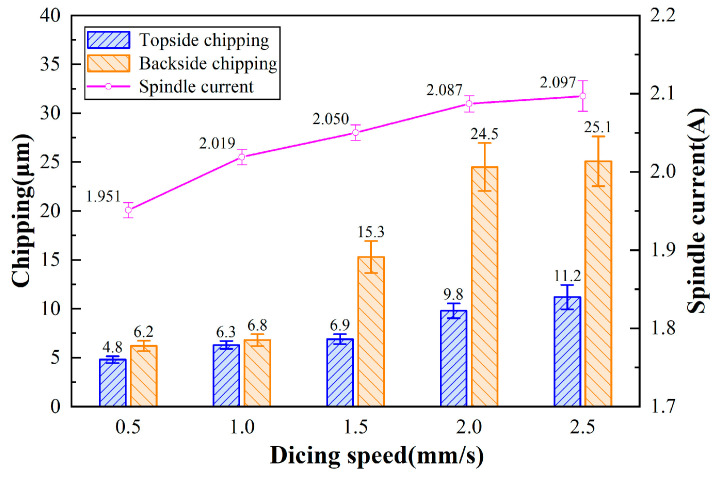
Effect of feed speed on chipping of 4H-SiC and spindle current (*a_p_* = 390 μm, *n* = 30,000 rpm).

Figure 10 illustrates the chipping micrographs of 4H-SiC cut by the dicing blade at different feed speeds. As shown in Figure 11, the effect of feed speed on the chipping of 4H-SiC and current is shown. As the feed rate increases, the spindle current becomes larger accordingly, and the top-back chipping size is positively correlated with the feed speed. The diamond abrasive grains involved in the cutting, as the feed rate increases, need to remove more material per rotation, so the stress applied to the dicing blade is greater. At the same time, more stress is applied to the workpiece, which leads to more severe chipping. Observing Figure 10(c-1), the size of the back chipping is 26 μm, which exceeds the thickness of the dicing blade by 23 μm, indicating that this feed rate is close to the critical value. Cutting quality performs better at lower feed speeds; however, this affects efficiency. In addition, when the feed speed is too low, the grinding force generated by cutting is small, which inhibits the self-sharpening performance of the dicing blade, and the passivated abrasive grains cannot be dislodged in time when cutting longer distances, resulting in a passivated dicing blade and a decrease in cutting quality.

#### 3.2.3. Effect of Spindle Speed of Cut on Chipping Quality

**Figure 12:**
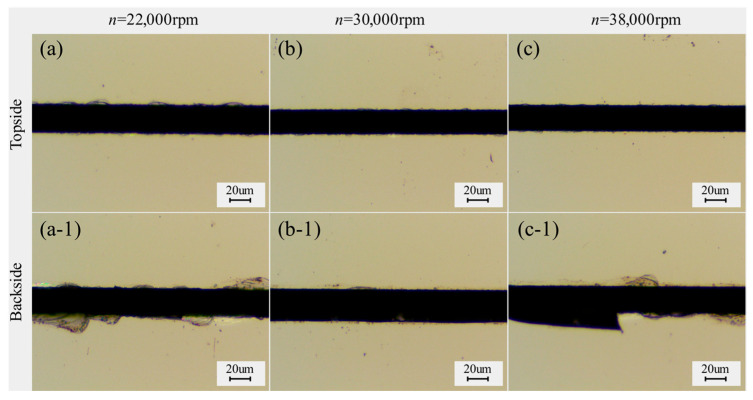
Chipping micrographs of 4H-SiC cut by dicing blade at different spindle speeds: (**a**–**c**) front, (**a-1**–**c-1**) back (*a_p_* = 390 μm, *V_w_* = 1 mm/s).

**Figure 13:**
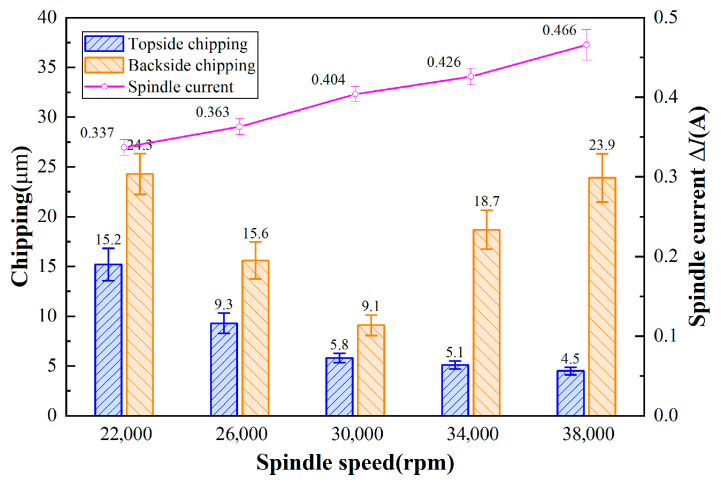
Effect of spindle speed on the chipping of 4H-SiC and the amount of change in spindle current (*a_p_* = 390 μm, *V_w_* = 1 mm/s).

With different spindle speeds, the power of the spindle is different, and the load generated by the cutting water on the blade will be different, so the difference in current between the cut workpiece and the non-cut workpiece at different spindle speeds is used to reflect the change in grinding force. Figure 12 shows the chipping micrographs of the dicing blade cutting 4H-SiC at different spindle speeds. As shown in Figure 13 shows the effect of spindle speed on chipping of 4H-SiC and the amount of change in spindle current. Front-side chipping decreases with increasing spindle speed. At high rotational speeds, the depth of abrasive grit cut into the workpiece decreases, and, therefore, less stress is applied to the workpiece. However, it was found that the increase in spindle speed was accompanied by an increase in the amount of change in spindle current, which may be attributed to reduced cooling and debris removal efficiency of the cutting water at high spindle speeds, increasing the cutting resistance. The back chipping decreases first and then increases, with the best performance at 30,000 rpm. The backside chipping decreases first due to the smaller depth of cut of the abrasive grain and then increases, probably due to the reduced access of the cutting water to the cutting area on the one hand, and the relatively higher vibration produced by the high spindle speed on the other hand. In addition, by choosing a lower spindle speed of 30,000 rpm, the force generated by the cut is greater and contributes to the self-sharpening mechanism of the dicing blade.

### 3.3. Effect of Abrasive Grain Size on Chipping Quality

**Figure 14:**
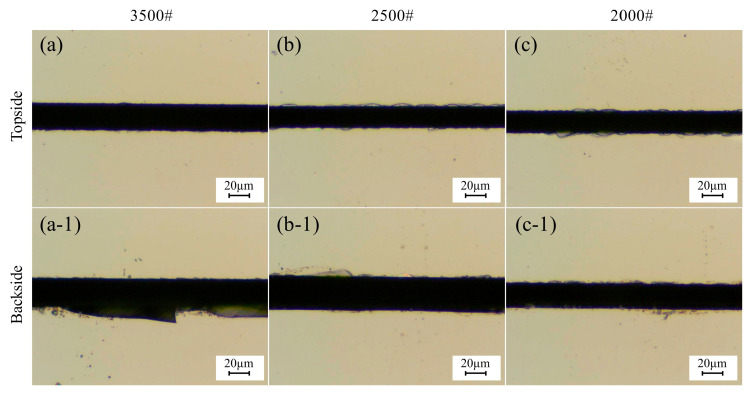
Chipping micrographs of 4H-SiC cut by dicing blades with different abrasive grain sizes: (**a**–**c**) top, (**a-1**–**c-1**) back (*a_p_* = 390 μm, *V_w_* = 1 mm/s, *n* = 30,000 rpm). # represents the mesh size.

**Figure 15:**
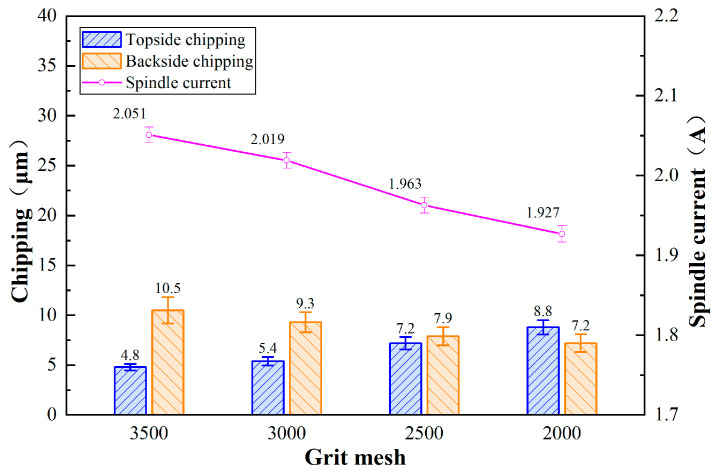
Effect of grit size on chipping of 4H-SiC and spindle current (*a_p_* = 390 μm, *V_w_* = 1 mm/s, *n* = 30,000 rpm).

Figure 14 shows micrographs of chipping of 4H-SiC cut by dicing blades with different abrasive grain sizes. Figure 15 shows the effect of abrasive grit size on the chipping of 4H-SiC with the amount of change in spindle current. It is found that as the abrasive grit size increases, the spindle current decreases. According to the grinding size effect, smaller grits have a smaller depth of cut and require relatively more energy per unit volume of material removed. In addition, larger grits have a greater depth of cut, which reduces the loss of sliding and plowing energy during material removal, resulting in a higher effective material removal rate and therefore a reduction in spindle current. The reason that the frontal chipping becomes larger with increasing abrasive grit size is due to the fact that larger abrasive grits collide with the workpiece and produce a greater impact, hence the chipping size becomes larger. However, the backside chipping was found to be smaller with increasing grit size, which is mainly attributed to the larger grit size of the dicing blade, which has a more pronounced grit projection height, creating more chip space, and the chips generated by the cut are more easily discharged from the cutting area, reducing the vertical force exerted on the backside of the workpiece.

### 3.4. Chipping Formation and Material Removal Mechanisms

**Figure 16:**
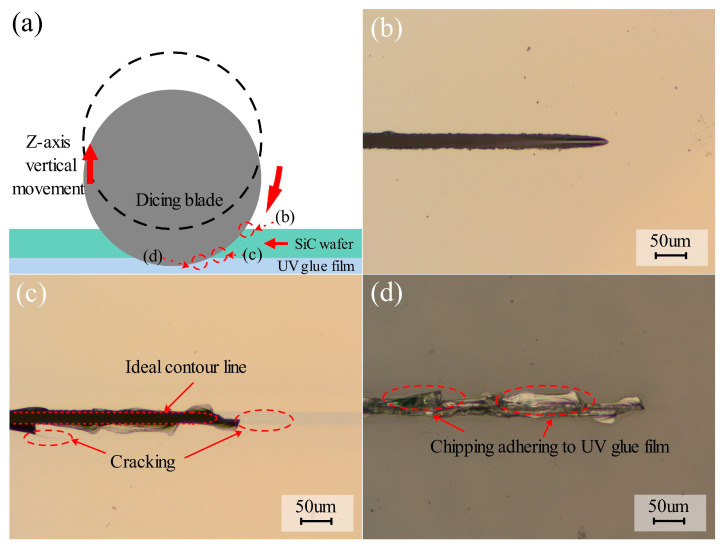
(**a**) Schematic diagram of the experimental principle and sampling area in the cutting contact arc area, (**b**–**d**) micrographs of 4H-SiC front chipping, back chipping, and UV film cut channel residue, respectively.

On the basis of the above process experiments, taking into account the quality of front-side chipping and back chipping and cutting efficiency, a set of parameters with the best cutting quality was selected, with a feed rate of 1 mm/s, a spindle speed of 30,000 rpm, a depth of cut of 0.390 mm, and an abrasive mesh number of 3000, and the experiments of cutting the contact arc zone were carried out. Figure 16a illustrates the schematic of the sampling area. The corresponding observational results are presented in Figure 16b–d. These micrographs provide direct visual evidence for the distinct material removal mechanisms discussed below:

1. Evidence for Front-Side Impact Mechanism: As shown in Figure 16b, the end of the cutting channel exhibits a smooth, continuous semi-circular arc profile that strictly follows the blade curvature. This feature indicates that the material removal on the front side is dominated by the high-speed impact and micro-cutting of abrasive grains.
2. Evidence for Backside Bending Fracture: In contrast, the backside morphology in Figure 16c displays a distorted, non-circular profile. Macroscopic cracks extending along the cutting direction are clearly visible (marked as “Cracking”). This visual phenomenon supports the conclusion that the backside material fractured unstably due to the bending stress concentration at the wafer bottom.
3. Evidence for Crush-Down Mechanism: Figure 16d clearly shows large 4H-SiC fragments embedded into the adhesive layer of the UV film (circled in red). This confirms that the brittle debris was pressed downwards by the blade into the soft film.

**Figure 17:**
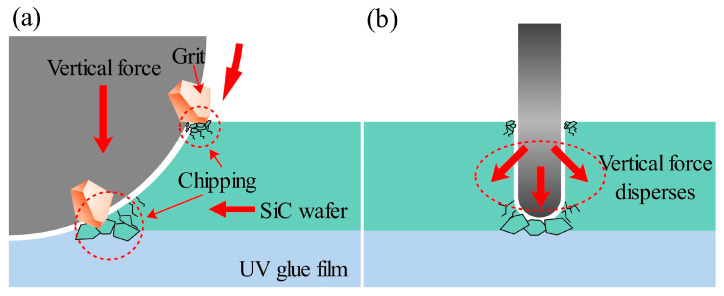
Mechanism of top-back chipping formation: (**a**) side view, (**b**) front view.

Therefore, it can be concluded that the frontal chipping was caused by the abrasive grains on the surface of the dicing blade hitting the 4H-SiC surface. Backside chipping is caused by cracks inside the workpiece. Specifically, when a carrier film with a cushioning effect is used to fix a hard and brittle material, the vertical force applied to the workpiece is relatively dispersed, and the cracks generated inside the workpiece become larger as the dicing blade moves closer to the carrier film. When the backside of the workpiece is crushed and breaks, backside chipping is formed. A diagram showing the mechanism of the formation of top-back chipping is shown in Figure 17.

It should be noted that the compliance and thickness of the UV carrier film play an important role in backside chipping formation. The compliant adhesive layer redistributes the vertical cutting load and allows subsurface cracks to propagate and coalesce before final separation. Variations in film thickness or adhesive stiffness are therefore expected to influence backside chipping behavior.

Removal patterns during cutting of brittle materials can be analyzed using threshold damage theory, and the critical depth of cut *d_c_* can be characterized using a modified brittle–plastic transition (BDS) model [32]:

(1) $$d_{c}\;=\;\beta (\frac{H}{E})(\frac{K_{c}}{H}{)}^{2}$$

where E is the modulus of elasticity, H is the hardness, and *K_c_* is the fracture toughness. In the C-face (0001) of 4H-SiC, the crystal direction $\left[11\bar{2}0\right]$. β is an empirical constant with a value of 0.15. The modulus of elasticity is 426 Gpa, the fracture toughness is 2.94 Mpa·m1/2, and the hardness is 37.0 Gpa [32]. Therefore, *d_c_* is calculated to be 16.2 nm using Equation (1).

**Figure 18:**
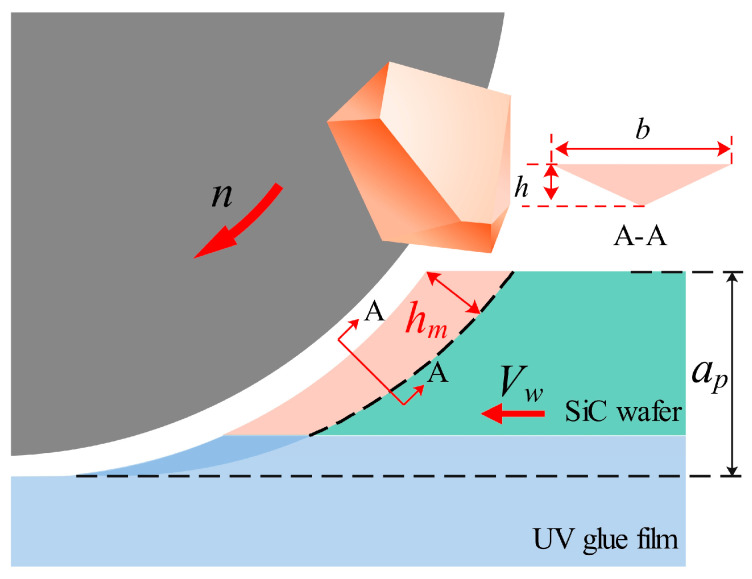
Schematic of material removal by a scribe knife fully cutting 4H-SiC.

Figure 18 shows a schematic of the material removal by the dicing blade fully cutting 4H-SiC. The dicing blade is kept rotating at high speed without displacement, the workpiece is fed from right to left, and the diamond abrasive grains remove material from the area of the workpiece that is in contact with the dicing blade, assuming that the diamond dicing blade is rigid, and *h_m_* can be determined from the geometrical configuration as follows [33]:

(2) $$h_{m}\;=[(4/Cr)(V_{w}/V_{s})(a_{p}/d_{s}{)}^{1/2}{\;]}^{1/2}$$

where *V_w_* is the feed rate, *V_s_* is the spindle linear speed, *a_p_* is the depth of cut, *d_s_* is the diameter of the dicing blade, *C* is the effective number of abrasive grains per unit area of the surface of the dicing blade, and *r* is the chip width/thickness ratio. In this experiment, the dicing blade used had an abrasive grain mesh of 3000 and a concentration of 70, usually the average spacing of abrasive grains after the preparation of this type of dicing blade is about 8–10 μm, assuming that the exposed abrasive grains on the surface of the dicing blade have a uniform distribution of 10 μm spacing and that the number of abrasive grains with an effective cutting edge accounts for 30% of the total number of exposed abrasive grains, so *C* is taken as 3000 grains/mm^2^. The shape of the abrasive grains used for cutting is tetrahedral with a sharp cutting edge. *r* is taken as 10 according to the existing research reports, and the shape of the abrasive grains used for cutting is tetrahedral with a sharp cutting edge. *V_w_* is 1 mm/s, *V_s_* is 88.467 m/s, *a_p_* is 0.390 mm, and *d_s_* is 56.320 mm. Therefore, the calculation gives the value of hm as 11.0 nm.

**Figure 19:**
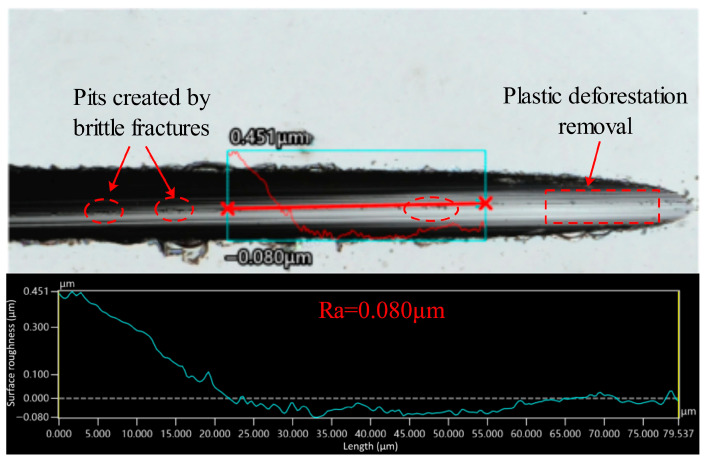
Surface morphology and roughness measurement results of cutting contact arc area.

**Figure 20:**
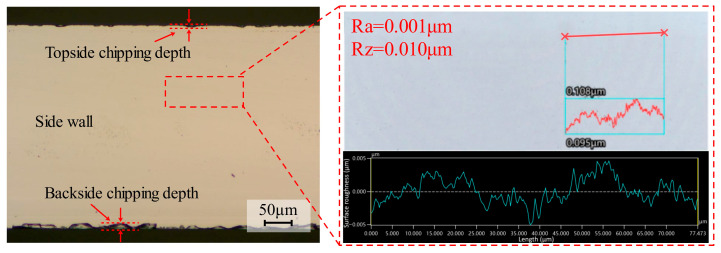
Surface morphology and roughness measurement results of the sidewalls.

The maximum undeformed chip thickness hm is 11.0 nm, which is smaller than the critical depth of cut thickness *d_c_*, which is 16.2 nm. Since *h_m_* (11.0 nm) is less than *d_c_* (16.2 nm), the cutting process tends to occur in the ductile regime during 4H-SiC cutting. The material removal mode can be characterized by examining the surface morphology of the cutting contact arc zone. As shown in Figure 19, the surface morphology and roughness measurements of the cut contact arc region under the above process parameters are shown. It was found that the surface of the cut contact arc area had a uniform distribution of grinding streaks and a surface roughness of 80 nm, which showed significant plastic flow characteristics. In addition, it was found that no obvious cracks existed on the surface, but a small number of craters formed by brittle damage were distributed, and the diameter of the craters was about 3 μm, which was attributed to the fact that the abrasive grains on the surface of the dicing blade were more or less randomly arranged, and the distribution of the height of the abrasive grain protrusion and the spacing of the successive cutting edges was not uniform, and abrasive grains were also dislodged in the process of cutting, which would result in the generation of these craters. In addition, the surface characteristics of the sidewalls can also reflect the pattern of material removal during the cutting process, with the formation of the sidewalls being largely attributable to the abrasive grains in the area where the sides of the dicing blade meet the end face, and the combined effect of the side abrasive grains on the workpiece. As shown in Figure 20, the surface morphology of the sidewall and the results of the roughness measurements are shown. The sidewalls are smooth, almost no grinding streaks are observed, and the surface roughness measurement is 1 nm in the area where there are no defects or pits in the sidewalls. The 4H-SiC surface roughness of 1 nm is to be achieved, which is usually obtained by fine grinding of the workpiece surface with a 3000 mesh grinding wheel in the silicon carbide substrate manufacturing process. Therefore, it can be concluded that the material is mainly plastically removed under these process conditions.

To further rationalize this observation, the material removal mechanism is discussed below by combining crack propagation theory with the brittle–plastic transition model. In this study, the material removal mechanism under the selected process parameters was analyzed in terms of stress distribution, crack initiation, and propagation behavior. While the experimental observation suggested that the material removal is primarily plastic, a more comprehensive explanation requires correlating the observed phenomena with crack propagation theory and a brittle-to-ductile transition model.

According to fracture mechanics, brittle fracture is driven by the propagation of pre-existing cracks when the stress intensity at the crack tip exceeds the critical fracture toughness $K_{c}$. However, under the high hydrostatic compressive stresses induced by the interaction between the dicing blade and SiC wafer, the stress state near the tool–workpiece interface tends to suppress crack propagation. This suppressive effect raises the effective threshold for brittle crack growth, which reduces the likelihood of catastrophic brittle removal under our cutting conditions.

In parallel, the brittle-to-ductile transition model proposed for hard and brittle materials suggests that when the depth of cut or the characteristic deformation zone remains below a critical value, plastic deformation mechanisms dominate over fracture mechanisms. In SiC, this transition depth $h_{c}$ can be estimated by comparing the material’s intrinsic fracture toughness and hardness (e.g., $h_{c}\propto (\frac{K_{c}}{H}{)}^{2}$), where $K_{c}$ is the fracture toughness, and H is the hardness. With the applied cutting depth and feed rate, the cutting process in this work remained near or below the predicted critical transition depth. As a result, the stress states and deformation conditions favored subsurface plastic flow and micro-plowing rather than the unstable propagation of sharp microcracks.

Therefore, the observed dominance of plastic material removal can be rationalized by the combined effects of (i) suppressed crack propagation due to high compressive stresses, and (ii) a material removal regime that lies within the brittle-to-ductile transition zone as dictated by fracture mechanics-based criteria. These interpretations aligned with the microstructural observations and chipping measurements presented previously.

## 4. Conclusions

This study provides a systematic experimental and mechanistic investigation of ultra-precision dicing of 4H-SiC wafers using a hub-type ultra-thin electroplated diamond blade. By combining spindle current-based process analysis, chipping characterization, and material removal modeling, the dynamic cutting behavior and the distinct formation mechanisms of front-side and backside chipping are clarified. The conclusions obtained are as follows:

1. The process of spindle current change for a single channel of 4H-SiC cut by the dicing blade can be divided into seven key points. Among them, the lowest point of the dicing blade occurs at contact with the workpiece. To the stage of completely cutting into the workpiece, the area of the contact arc does not change, the spindle current continues to increase, and the growth rate slows down, which is attributed to significant interference between the blade sidewall and the workpiece.
2. As spindle speed increases, the average value of the size of the front-side chipping decreases, while the average value of the size of the back avalanche decreases and then increases. The first decrease is due to the depth of cut of a single abrasive grain to reduce the stress applied to the workpiece, and then increased on the one hand, which may be due to the high-speed cutting water into the cutting area of the opportunity to reduce on the other hand, as the vibration generated by a high spindle speed is relatively greater. The abrasive grain size is directly proportional to the front-side chipping size and inversely proportional to the backside chipping size. This is because larger abrasive grains have a more pronounced protrusion height, creating larger chip pockets. Consequently, chips are more easily discharged from the cutting zone, reducing the vertical force exerted on the back of the workpiece.
3. The formation mechanism of front-side chipping involves the impact of the diamond grits with the workpiece, while the mechanism for the formation of back chipping is caused by the cracks generated inside the workpiece. In the case of using the optimal process parameters, the calculated maximum undeformed chip thickness is 11.0 nm, which is lower than the calculated critical chip depth of 16.2 nm. Uniform grinding marks and pronounced plastic flow characteristics were observed on the cutting contact arc surface, and the material removal process is dominated by plastic deformation.

## Figures and Tables

**Table 1:** Specifications of dicing blades.

	3000-R-70 DCB
Outer diameter (mm)	56.320
Bond	Nickel bond
Grit concentration	70
Avg. grit size	Grade 3000 (4.5–5.5 μm)
Blade exposure(μm)	660
Blade thickness(μm)	23

**Table 2:** Conditions of dicing process.

Conditions	Feature
Incomplete cutting	*a_p_*: 80, 150, 220, 290, 360 μm
Complete cutting	*a_p_*: 370, 380, 390, 400, 410 μm
	*V_w_*: 0.5, 1, 1.5, 2, 2.5 mm/s
	*n*: 22,000, 26,000, 30,000, 34,000, 38,000 rpm

## Data Availability

The original data supporting this study is included in the article.
